# Both hemispheric influenza vaccine recommendations would have missed near half of the circulating viruses in Madagascar

**DOI:** 10.1111/irv.12517

**Published:** 2017-11-28

**Authors:** Julia Guillebaud, Jean‐Michel Héraud, Norosoa H. Razanajatovo, Alicia A. Livinski, Wladimir J. Alonso

**Affiliations:** ^1^ National Influenza Centre Virology Unit Institut Pasteur de Madagascar Antananarivo Madagascar; ^2^ National Institutes of Health Library Office of Research Services, Office of Management National Institutes of Health Bethesda MD USA; ^3^ Laboratory for Human Evolutionary and Ecological Studies Institute of Biosciences University of São Paulo São Paulo Brazil

**Keywords:** influenza, Madagascar, match, seasonality, timing, vaccination

## Abstract

**Background:**

Influenza immunization still poses a critical challenge globally and specifically for tropical regions due to their complex influenza circulation pattern. Tropical regions should select the WHO's Northern Hemisphere or Southern Hemisphere recommended vaccine composition based on local surveillance. Analyses of influenza immunization effectiveness have neglected to account for the proportion of circulating viruses prevented from causing infection each year. We investigate this question for Madagascar, where influenza vaccines are not widely available.

**Methods:**

Seventy‐eight Malagasy influenza strains characterized from 2002 to 2014 were challenged with hypothetical scenarios in which the WHO's Northern Hemisphere and Southern Hemisphere recommended vaccine compositions were provided to the population. Match between circulating and vaccine strains was determined by haemagglutination inhibition assays. Strain‐specific positive matches were scored assuming 9 months of protection, and scenarios incorporated vaccine delays from zero to 5 months.

**Results:**

Malagasy influenza strains matched 54% and 44%, respectively, with the Northern Hemisphere and Southern Hemisphere recommended vaccine strains when the vaccine was delivered as soon as available. The matching values further decreased when additional delivery and application delays were considered. Differences between recommended compositions were not statistically significant.

**Conclusion:**

Our results showed matching with the Northern Hemisphere vaccine barely above 50%, even in the more favourable scenario. This suggests that if implemented, routine influenza vaccines would not provide an optimal protection against half of the influenza strains circulating in any epidemic season of Madagascar. We suggest that this limitation in influenza vaccine efficacy deserves greater attention, and should be considered in cost/benefit analyses of national influenza immunization programmes.

## 1 BACKGROUND

Influenza's ability to antigenically drift imposes the constant need for an update to the annual World Health Organization's (WHO) recommended vaccine composition for both the Northern Hemisphere and Southern Hemisphere (NH and SH). The WHO's recommended vaccine composition includes three strains of influenza virus: two influenza A and one influenza B.^1^ The variable level of putative cross‐protection that can be obtained from non‐matching circulating strains^2^ allows for some protection even when the vaccine compositions do not match circulating viruses. Yet, the proportion of circulating influenza viruses in the epidemic period after vaccination that matches with the recommended vaccine composition is not a metric used to measure potential vaccine protection.

Tropical countries have a much more complex circulation pattern of influenza^3^, ^4^ as they are frequently out of phase with the dynamics predicted for their hemispheric group. Several studies have assessed the best recommended vaccine composition mainly for the Southern Hemisphere.^4^, ^5^, ^6^, ^7^, ^8^, ^9^ Studies by Mello et al,^5^ Waiboci et al,^9^ Alonso et al^10^ and Mah‐E‐Muneer et al^11^ used a method to measure the proportion of circulating influenza viruses in the epidemic period after vaccination that matched with the recommended vaccine compositions in tropical countries. Although not the objective and not discussed in the above studies, by reviewing their data we observed that their rates of vaccine matching were low (below 50%). This raises concerns about vaccine protection and implementation of successful immunization programmes.

Madagascar is unique as it is an island located in the tropical belt of the Southern Hemisphere. It has a tropical climate with two different seasons: hot and humid from November to April, and cool and dry from May to October. However, larger climatic variations are encountered within the island. Madagascar has a robust influenza surveillance system, and the absence of an influenza national vaccination programme provided an opportunity to investigate the importance of vaccine matching to circulating strains. In Madagascar, influenza vaccine is available only at some private institutions (eg, Institut Pasteur de Madagascar) and is not widely available to the general population. Additionally, these analyses may assist Malagasy health authorities to identify the optimal timing and vaccine formulation in the event a population‐wide routine influenza immunization programme is implemented.

## 2 METHODS

### 2.1 Influenza strain characterization

Madagascar has an effective clinical and virological influenza surveillance system allowing for robust multi‐year epidemiological studies.^12^, ^13^, ^14^ Influenza surveillance relies on the participation of sentinel sites sharing clinical information on a weekly basis^12^, ^15^ and on specimens collected throughout the year. An influenza epidemic is arbitrarily defined by the National Influenza Centre (NIC) of Madagascar as an increase in influenza positivity rate of 50% of all sentinel sites. Nasopharyngeal and/or throat swabs from patients with influenza‐like illness (ILI) (as defined by the evolving WHO case definition) are analysed as previously described.^12^, ^16^ Between 2002 and 2014, 4413 influenza viruses circulating in Madagascar were detected (for more details, see Alonso et al^7^ and Table S2). As part of the WHO Global Influenza Surveillance and Response System (GISRS), at least twice a year the NIC of Madagascar shares influenza virus isolates with the WHO Collaborative Centre (WHO CC) of London^17^, ^18^ where genetic and antigenic characterizations (HI assays) are performed for the update to the influenza vaccine recommendations. Seventy‐eight samples (of 4413) were selected for our analyses based on their representativeness of the viruses circulating in different parts and times (start, middle and end of epidemics) of the country, circulating types and/or subtypes, and the availability of genetic and antigenic characterizations.^19^

### 2.2 Matching of vaccine strains according to different scenarios, 2002‐2014

The method used to evaluate and compare alternative vaccination recommendations both in terms of time and composition (NH vs SH) was developed by one of the authors (WJA) and described elsewhere.^5^ In brief, we analysed the hypothetical matching success that each hemispheric WHO's influenza recommended vaccine composition (Table S1) would have had soon after vaccination against Malagasy influenza viruses circulating from 2002 to 2014. Strain‐specific positive matches were scored when circulating strains overlapped with the protective period (heuristically chosen as 9 months) and following the hypothetical vaccination with the same strain. Malagasy influenza A circulating strains were matched with vaccine strains if they belonged to the same subtype and were antigenically similar in the HI assay. Malagasy influenza B strains were matched with vaccine strains if they belonged to the same lineage and were antigenically similar to the vaccine strain in the HI assay.

Next, the vaccine match for each year and in total was estimated, according to six vaccine delivery scenarios:

1. Scenario Zero: No lag from the time that the vaccine was available for both NH (October) and SH (April) recommendations;^5^
2. Scenarios One to Five: Lags that could occur (eg, logistical issues) leading to later vaccination timing (up to 5 months).^11^

### 2.3 Statistical analysis

Global comparison between WHO's NH and SH recommended vaccine composition scenarios was performed by comparing matching proportions at each delivery lag (zero to 5 months) independently. To allow all years to contribute equally in the analyses, the grand total proportion of matching success was the average of the matching success of each year. Differences in proportions were assessed in chi‐squared tests using Stata Statistical Software: Release 13 (StataCorp LP, College Station, TX, USA). Values of *P* < .05 were considered significant.

## 3 RESULTS

### 3.1 Detection of influenza strains

**Figure 1:**
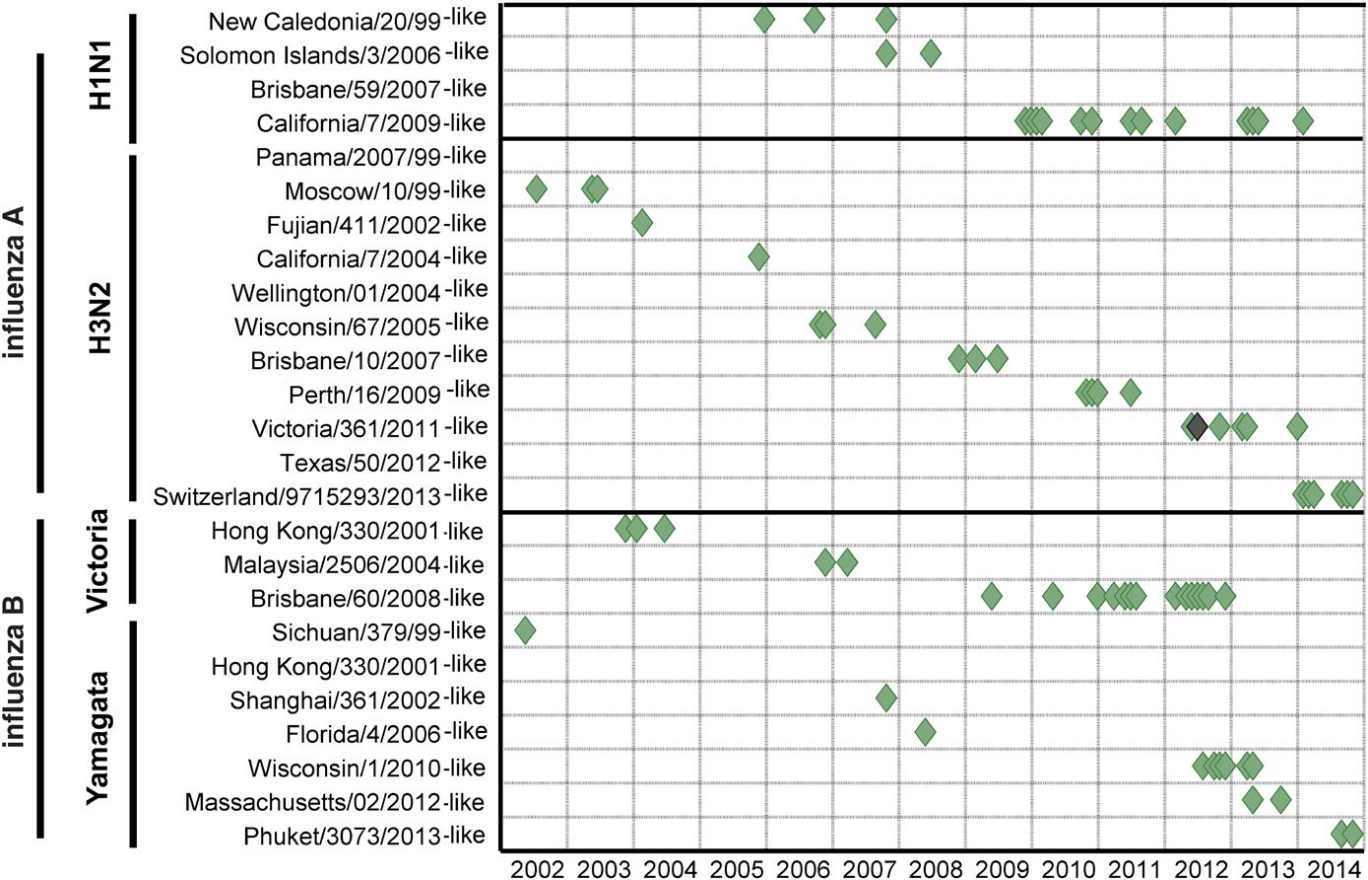
Monthly detection of influenza viruses isolated in Madagascar named according to their similarity to vaccine influenza strain. Apart from one instance when there were two detections of the same strain in a single month (darker symbol at A(H3N2)Victoria/361/2011‐like), a diamond represents a single detection per month

**Figure 2:**
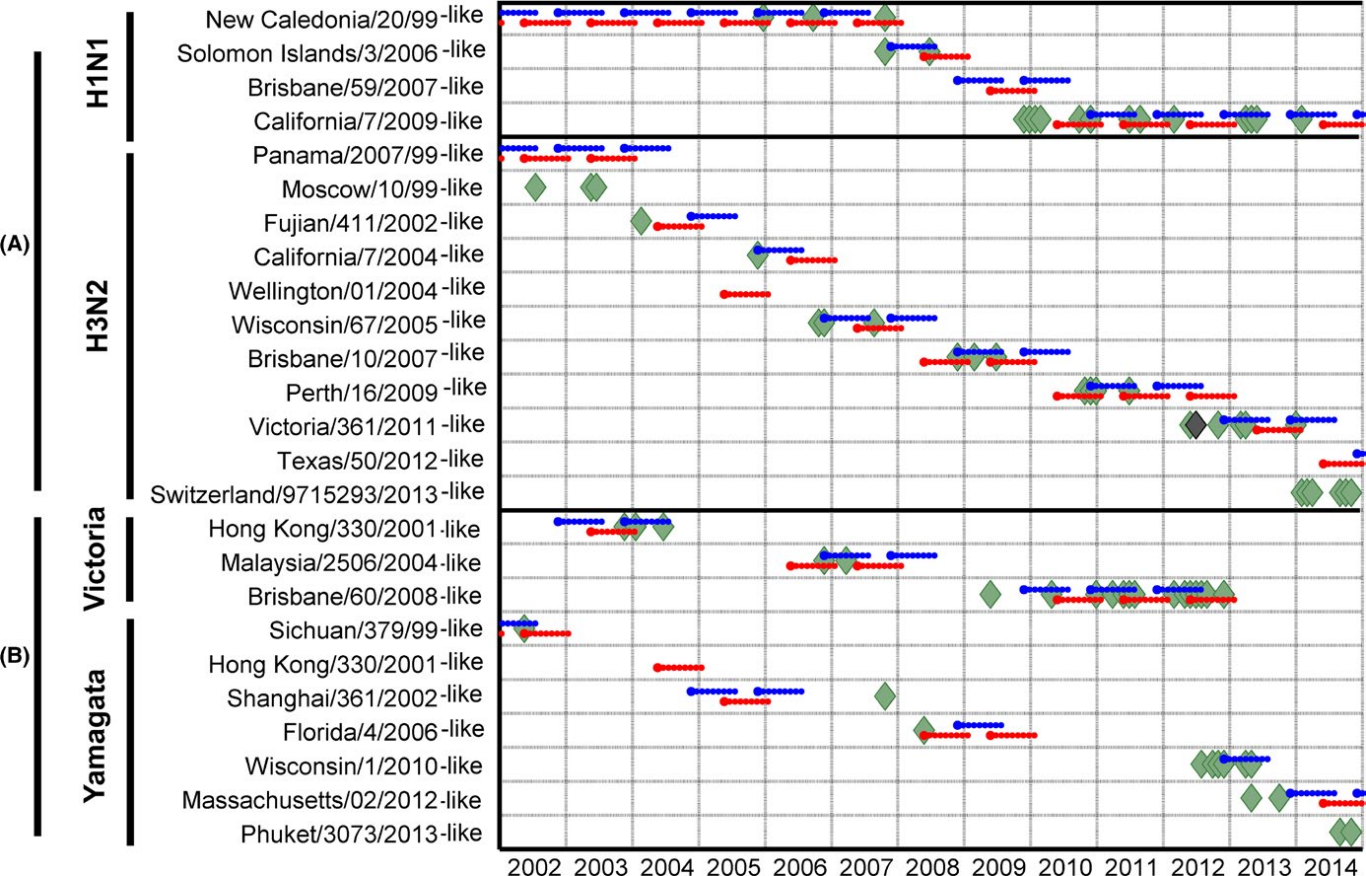
Matching success of recommended vaccine strains against influenza viruses isolated monthly in Madagascar. Blue and red lines correspond, respectively, to WHO's NH and SH vaccine recommendations and schedule (assumed 9 subsequent months of immunity protection). The rate of successful matches between the vaccines and circulating influenza strains is quantified by the overlap between the simulated vaccine immunization period (blue or red) and actual virus isolations (green diamonds) through this period. Diamonds represent a single detection per month, with exception of the darker one which represent two detections in that month (Fig. 1)

The 78 Malagasy viruses analysed included: 18 A/H1N1 (13 being of the 2009 pandemic strain); 28 A/H3N2; 19 B/Victoria; and 13 B/Yamagata. The results of the vaccine‐like characterization of each virus in reference to the timing of their respective collection are presented in Figure 1. All strains included in the analyses were either part of the WHO's influenza recommended vaccine composition for one or both hemispheres (at least once during the considered period 2002 to 2014) and/or detected in Madagascar during the same time period (Figures 1 and 2). Among the included vaccine strains, 3 influenza A strains (close to Brisbane/59/2007, Panama/2007/99 and Wellington/01/2004) were not detected in Madagascar, while A/H3N2/Moscow/10/99‐like and A/H3N2/Switzerland/2013‐like were detected in Madagascar, but were not part of any WHO's recommended vaccine composition. The final analysis was performed on 78 viruses as 2 influenza B strains (close to Shanghai/361/2002 in 2004 and Malaysia/2506/2004 in 2007) were excluded because the exact month of collection was unknown.

### 3.2 Matching success of both vaccine strategies (NH vs SH) without vaccine delivery delay

Figure 2 illustrates the analyses performed for the NH and SH vaccines for only one hypothetical scenario for each hemispheric recommended vaccine composition: if vaccines were delivered as soon as available after the WHO's recommendations were issued. Table 1 and Figure S1 show the number and proportion of detected viruses of the final data set that hypothetically matched with either NH or SH recommended vaccine strains per year. When no lag in vaccine delivery was considered (central columns of the table), 40 and 30 samples matched with NH and SH vaccine recommendations, respectively. In years 2002, 2003, 2006 and 2011, both NH and SH recommended vaccine compositions had the same level of successful matching of 50%, 33%, 50% and 86%, respectively.

**Table 1:** Matching success of vaccine recommendations against strains of influenza viruses isolated monthly from 2002 to 2014 (n = number of viruses) according to various vaccine delivery time lag scenarios

Lags (months)												
	Southern hemisphere vaccination						Northern hemisphere vaccination					
	−5	−4	−3	−2	−1	0	0	−1	−2	−3	−4	−5
2014 (n = 9)	‐	‐	‐	‐	‐	‐	1 (11)	1 (11)	1 (11)	‐	‐	‐
2013 (n = 10)	4 (40)	4 (40)	3 (30)	2 (20)	1 (10)	1 (10)	8 (80)	8 (80)	7 (70)	7 (70)	6 (60)	4 (40)
2012 (n = 16)	6 (38)	5 (31)	5 (31)	5 (31)	6 (38)	5 (31)	7 (44)	7 (44)	7 (44)	7 (44)	6 (38)	6 (38)
2011 (n = 7)	5 (71)	2 (29)	2 (29)	3 (43)	5 (71)	6 (86)	6 (86)	7 (100)	7 (100)	7 (100)	7 (100)	6 (86)
2010 (n = 9)	5 (56)	6 (67)	6 (67)	6 (67)	6 (67)	6 (67)	5 (56)	3 (33)	1 (11)	1 (11)	1 (11)	2 (22)
2009 (n = 5)	2 (40)	1 (20)	1 (20)	1 (20)	2 (40)	1 (20)	2 (40)	2 (40)	2 (40)	2 (40)	1 (20)	1 (20)
2008 (n = 3)	1 (33)	1 (33)	1 (33)	1 (33)	2 (67)	3 (100)	2 (67)	1 (33)	1 (33)	1 (33)	1 (33)	1 (33)
2007 (n = 5)	2 (40)	2 (40)	3 (60)	3 (60)	2 (40)	2 (40)	1 (20)	2 (40)	2 (40)	3 (60)	3 (60)	2 (40)
2006 (n = 4)	1 (25)	2 (50)	2 (50)	2 (50)	2 (50)	2 (50)	2 (50)	‐	1 (25)	1 (25)	1 (25)	1 (25)
2005 (n = 2)	1 (50)	1 (50)	1 (50)	1 (50)	1 (50)	1 (50)	2 (100)	1 (50)	‐	‐	‐	1 (50)
2004 (n = 3)	2 (67)	1 (33)	1 (33)	1 (33)	1 (33)	1 (33)	2 (67)	2 (67)	2 (67)	1 (33)	1 (33)	1 (33)
2003 (n = 3)	1 (3)	1 (33)	1 (33)	1 (33)	1 (33)	1 (33)	1 (33)	‐	‐	‐	1 (33)	1 (33)
2002 (n = 2)	1 (50)	1 (50)	‐	‐	‐	1 (50)	1 (50)	1 (50)	1 (50)	1 (50)	1 (50)	1 (50)
**Total**	31 (42)	27 (37)	26 (34)	26 (34)	29 (38)	30 (44)	40 (54)	35 (42)	32 (38)	31 (36)	29 (36)	27 (36)

The vaccination matching for each year (% in parenthesis) and in total, considered that no lag would exist from the time that the vaccine was made available for each hemisphere (two columns in the centre) and time lags of up to 1, 2, 3, 4 and 5 months (eg, due to logistical problems). Blue and red colours correspond, respectively, to WHO's NH and SH vaccine recommendations and schedules.

For the no lag scenario, the average of proportions was 54% and 44%, respectively, for matching the NH and SH recommendations. This indicated that the NH recommendation corresponded better to Malagasy strains circulation and timing; however, no statistical difference was observed (chi‐square test, *P* = .2). When looking at the strain type/subtype level, again no statistical difference was observed between NH and SH recommendations although the NH recommendations had a slightly higher match with the circulating Malagasy strains. Interestingly, influenza B viruses belonging to the Victoria lineage better matched to both vaccine recommendations than the other types or subtypes and had a higher rate of successful matching with the NH recommendations than the SH (84% vs 63%, see Table S3).

### 3.3 Matching success according to different vaccine delay scenarios

Results of analyses utilizing delays of 1‐5 months are summarized in Table 1 and Figure S1. Matching success was year‐dependent, but no specific pattern emerged. However, large variations in rates of successful matching were observed and ranged from zero to 100%. For a delay of up to 3 months, matching success was slightly higher for the NH recommendations than for the SH (36% and 34%, respectively). Further with a delay of up to 5 months, the SH recommendation (42%) was a slightly more successful match than for the NH (36%).

## 4 DISCUSSION

Our analyses showed that neither of the WHO's hemispheric influenza recommended vaccine compositions would have afforded good protective immunity to the Malagasy population against the strains circulating between 2002 and 2014. It is important to note that this result assumes full protection of vaccinated individuals despite the known difficulties to achieving complete immune response to the vaccine.^20^, ^21^ During any epidemic season and independent of the WHO's recommended vaccine composition, the match with the circulating strains in Madagascar would have been barely above 50% — even in the more favourable vaccine delay scenarios. We are confident that we did not underestimate the ability of historical vaccines to protect against circulating strains; in fact, if rare novel strains (that the surveillance system did not capture) were circulating during the years analysed, the match of the hemispheric recommended vaccine strains would have been even lower in our analyses (as vaccines are based on strains identified that are 6 months or older).

Our assessment may appear applicable only to Madagascar due to its erratic influenza viral circulation pattern.^7^ However, we found that low successful strain match between WHO's recommended vaccination compositions and circulating viruses is a constant, not an exception as based on a review of the data from studies that investigated the proportion of influenza strain matching and which took into account the timing of vaccine delivery.^4^, ^5^, ^9^, ^11^ Successful matching between NH and SH recommended vaccine strains and circulating viruses was reported at 39% and 40% in Bangladesh;^11^ 53.6% and 46.4% in Kenya;^9^ 18% and 35%^5^ and less than 30% and 20% for H3N2^4^ in Brazil. In temperate areas, studies focused mainly on influenza vaccine effectiveness (VE) solely. However, some previous works^22^, ^23^, ^24^ including also antigenic match data highlighted that vaccine mismatch can strongly impact on VE despite substantial cross‐protection. They suggested that antigenic characterization data should be included in VE estimates studies.

Our sample size might appear as a possible limitation of this study with 78 viruses over a period of 12 years. Our study is based on retrospective analyses on data obtained for other purposes (the global efforts of influenza surveillance for assisting the formulation of the vaccine by the WHO). Still, it is important to note that our sample size is double the number presented by the study that triggered the current WHO's attention on the problem of the vaccine composition and timing in the tropics.^5^

Regarding the optimal timing of influenza vaccination and the optimal composition recommendation to be adopted, our study showed that there would be no difference in Madagascar between the two WHO's hemispheric recommended vaccine compositions even when potential delays in vaccine availability after the issuance of the WHO's recommendations and its distribution were taken into consideration. The complexity of successfully matching influenza vaccination recommendations in tropical regions to circulating viruses is significant and needs further study.

National immunization programmes need to consider that routine influenza vaccinations might not protect their population from up to half of the circulating viruses in any epidemic influenza season. Vaccine delivery delays are not uncommon, and a delay may decrease the vaccine's ability to match the circulating viruses. To our knowledge, this facet of the rate of successful matching between the WHO's hemispheric recommended vaccine compositions and circulating viruses has not been previously discussed or studied in depth. We believe that this is a key factor, which needs to be considered for inclusion in cost‐effectiveness analyses of national immunization programmes.

## CONFLICT OF INTEREST

None declared.
