# Supplementary material for: Both hemispheric influenza vaccine recommendations would have missed near half of the circulating viruses in Madagascar
Source: Influenza Other Respir Viruses. 2017 Nov 28;11(6):473–8. doi: 10.1111/irv.12517 (PMC5705694; doi:10.1111/irv.12517)

**Supplementary Table 1: WHO influenza recommended vaccine compositions for the Northern and Southern Hemispheres from 2002 to 2014.** Antigenic characterization of the strains recommended for inclusion in the trivalent vaccine, by hemisphere, influenza season, and subtype.

| **WHO hemispheric recommendation** | **Season** | **A/H3N2** | **A/H1N1** | **B** |
| --- | --- | --- | --- | --- |
| NH | 2001-2002 | A/Panama/2007/99 | A/New Caledonia/20/99 | B/Sichuan/379/99 |
| SH | 2002 | A/Panama/2007/99 | A/New Caledonia/20/99 | B/Sichuan/379/99 |
| NH | 2002-2003 | A/Panama/2007/99 | A/New Caledonia/20/99 | B/Hong Kong/330/2001 |
| SH | 2003 | A/Panama/2007/99 | A/New Caledonia/20/99 | B/Hong Kong/330/2001 |
| NH | 2003-2004 | A/Panama/2007/99 | A/New Caledonia/20/99 | B/Hong Kong/330/2001 |
| SH | 2004 | A/Fujian/411/2002 | A/New Caledonia/20/99 | B/Hong Kong/330/2001 |
| NH | 2004-2005 | A/Fujian/411/2002 | A/New Caledonia/20/99 | B/Shanghai/361/2002 |
| SH | 2005 | A/Wellington/1/2004 | A/New Caledonia/20/99 | B/Shanghai/361/2002 |
| NH | 2005-2006 | A/California/7/2004 | A/New Caledonia/20/99 | B/Shanghai/361/2002 |
| SH | 2006 | A/California/7/2004 | A/New Caledonia/20/99 | B/Malaysia/2506/2004 |
| NH | 2006-2007 | A/Wisconsin/67/2005 | A/New Caledonia/20/99 | B/Malaysia/2506/2004 |
| SH | 2007 | A/Wisconsin/67/2005 | A/New Caledonia/20/99 | B/Malaysia/2506/2004 |
| NH | 2007-2008 | A/Wisconsin/67/2005 | A/Solomon Islands/3/2006 | B/Malaysia/2506/2004 |
| SH | 2008 | A/Brisbane/10/2007 | A/Solomon Islands/3/2006 | B/Florida/4/2006 |
| NH | 2008-2009 | A/Brisbane/10/2007 | A/Brisbane/59/2007 | B/Florida/4/2006 |
| SH | 2009 | A/Brisbane/10/2007 | A/Brisbane/59/2007 | B/Florida/4/2006 |
| NH | 2009-2010 | A/Brisbane/10/2007 | A/Brisbane/59/2007 | B/Brisbane/60/2008 |
| SH | 2010 | A/Perth/16/2009 | A/California/7/2009 | B/Brisbane/60/2008 |
| NH | 2010-2011 | A/Perth/16/2009 | A/California/7/2009 | B/Brisbane/60/2008 |
| SH | 2011 | A/Perth/16/2009 | A/California/7/2009 | B/Brisbane/60/2008 |
| NH | 2011-2012 | A/Perth/16/2009 | A/California/7/2009 | B/Brisbane/60/2008 |
| SH | 2012 | A/Perth/16/2009 | A/California/7/2009 | B/Brisbane/60/2008 |
| NH | 2012-2013 | A/Victoria/361/2011 | A/California/7/2009 | B/Wisconsin/1/2010 |
| SH | 2013 | A/Victoria/361/2011 | A/California/7/2009 | B/Wisconsin/1/2010 |
| NH | 2013-2014 | A/Victoria/361/2011 | A/California/7/2009 | B/Massachusetts/2/2012 |
| SH | 2014 | A/Texas/50/2012 | A/California/7/2009 | B/Massachusetts/2/2012 |

**Abbreviations:**

NH = WHO Northern hemisphere influenza recommended vaccine composition

SH = WHO Southern hemisphere influenza recommended vaccine composition

**Supplementary Table 2: Number of influenza viruses detected between 2002 and 2014 at the NIC of Madagascar.** Number of influenza viruses detected at the NIC of Madagascar from 2002 to 2014 both by isolation and RT-PCR.

|  | **2002** | **2003** | **2004** | **2005** | **2006** | **2007** | **2008** | **2009** | **2010** | **2011** | **2012** | **2013** | **2014** | Total |
| --- | --- | --- | --- | --- | --- | --- | --- | --- | --- | --- | --- | --- | --- | --- |
| **B** | 101 | 4 | 14 | 61 | 10 | 98 | 3 | 166 | 41 | 260 | 248 | 230 | 201 | 1437 |
| **A(H3N2)** | 10 | 75 | 67 | 43 | 155 | 4 | 64 | 27 | 7 | 167 | 223 | 16 | 368 | 1226 |
| **A(H1N1)** | - | - | - | 54 | 55 | 85 | 91 | 16 | 143 | - | - | - | - | 444 |
| **A(H1N1)pdm09** | - | - | - | - | - | - | - | 937 | 86 | 85 | 39 | 135 | 24 | 1306 |
| Total | 111 | 79 | 81 | 158 | 220 | 187 | 158 | 1146 | 277 | 512 | 510 | 381 | 593 | 4413 |

**Supplementary Table 3: Matching success of recommended vaccine strains per type/subtype of influenza strains from 2002 to 2014.** Number of matching strains (both in terms of composition and timing of vaccine delivery) are indicated and the corresponding proportion is in parenthesis.

|  | **H1N1 (n=18)** | | **H3N2 (n=28)** | | **B/VIC (n=19)** | | **B/YAM (n=13)** | |
| --- | --- | --- | --- | --- | --- | --- | --- | --- |
|  | SH | NH | SH | NH | SH | NH | SH | NH |
| **2014** | - | 1 | - | - | - | - | - | - |
| **2013** | - | 3 | 1 | 3 | - | - | - | 2 |
| **2012** | - | 1 | - | - | 5 | 5 | - | 1 |
| **2011** | 2 | 1 | 1 | 1 | 3 | 4 | - | - |
| **2010** | 2 | 1 | 3 | 2 | 1 | 2 | - | - |
| **2009** | - | - | 1 | 2 | - | - | - | - |
| **2008** | 1 | 1 | 1 | 1 | - | - | 1 | - |
| **2007** | 1 | - | 1 | - | - | 1 | - | - |
| **2006** | 1 | - | - | 1 | 1 | 1 | - | - |
| **2005** | 1 | 1 | - | 1 | - | - | - | - |
| **2004** | - | - | - | - | 1 | 2 | - | - |
| **2003** | - | - | - | - | 1 | 1 | - | - |
| **2002** | - | - | - | - | - | - | 1 | 1 |
| **Total** | 8 (%44) | 9  (%50) | 8 (%29) | 11 (%39) | 12 (%63) | 16 (%84) | 2 (%15) | 4 (%31) |

**Abbreviations:**

NH = WHO Northern hemisphere influenza recommended vaccine composition

SH = WHO Southern hemisphere influenza recommended vaccine composition

**Supplementary Fig. 1: Proportion of vaccination matching according to various vaccine delivery time lag scenarios.** Matching of vaccination for each year (and total), considered that no lag would exist from the time that vaccine was available for each hemisphere (two columns in the center) and considered time lags of up to 1, 2, 3, 4, and 5 months (due to logistical problems).


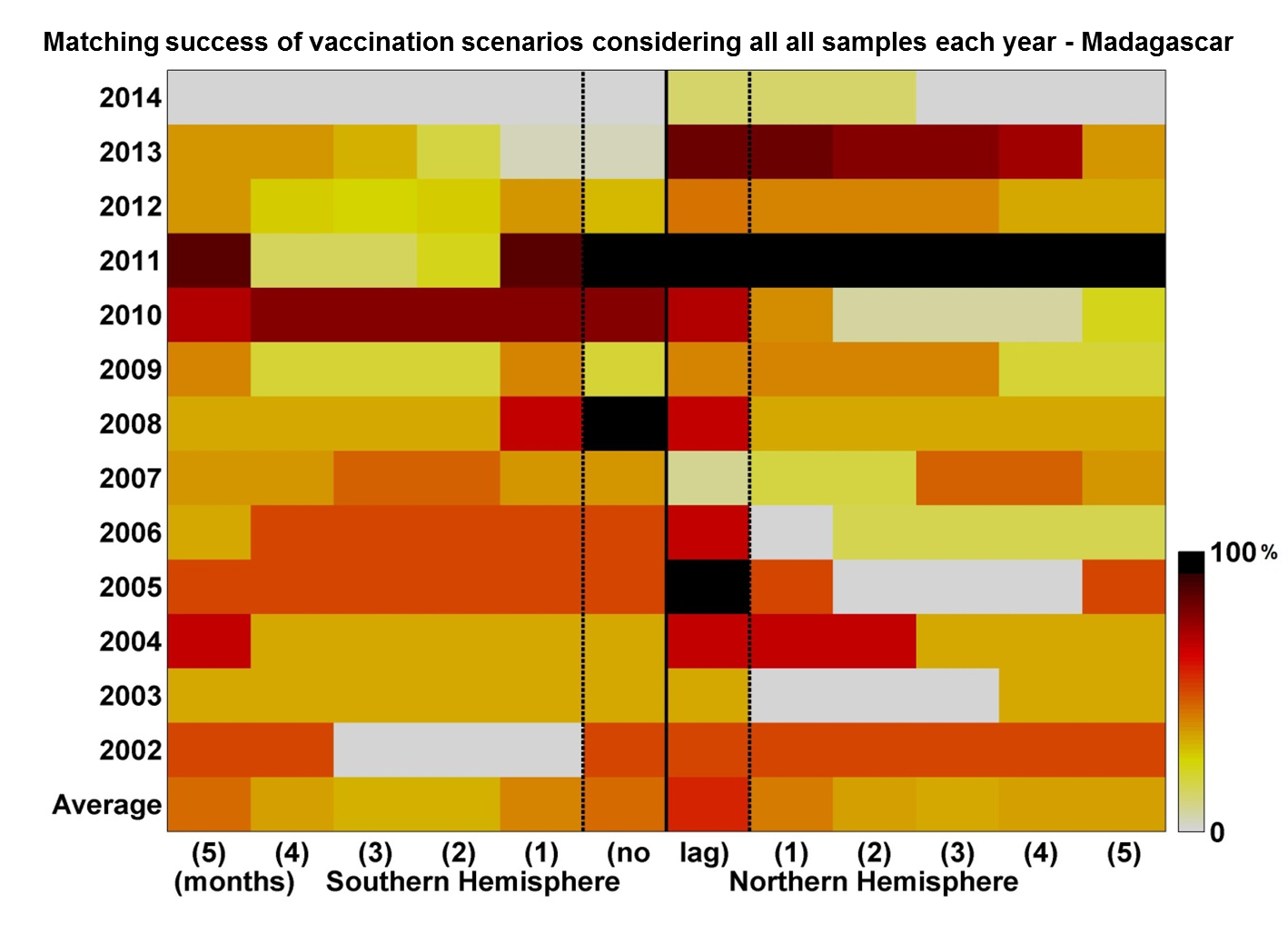

Supplement: Supplementary file 1 [file IRV-11-473-s001.doc]
